# A call for constructing a metric for monitoring CA-MRSA and other infectious diseases

**DOI:** 10.1186/s13690-020-0394-5

**Published:** 2020-03-18

**Authors:** Alaa Al Amiry

**Affiliations:** College of Pharmacy and Health Sciences, Ajman University, Al Jurf 2, POBox 346, Ajman, United Arab Emirates

**Keywords:** CA-MRSA, Infectious diseases, Metric, Value-based measurement, Health policy

## Abstract

The use of metrics is necessary for decision making when it comes to evaluating the need for interventions and improve healthcare delivery (Adams, Metrics: What Counts in Global Health, 2016). They inform policy makers on the scale of the problem in hand, and whether it’s time to intervene. It quantifies the matter of interest and gives an indication whether the intervention taken was effective or not- as a means for monitoring and evaluation (Murray and Frenk, Lancet 371:1191-1199, 2008).

This article advocates for a new metric by suggesting criteria to construct it, in order to provide a “value-based” measurement for CA-MRSA as a starting point for all infectious diseases. It also discusses challenges to this suggested approach.

## Background

The use of metrics is necessary for decision making when it comes to evaluating the need for interventions and improve healthcare delivery [1]. They inform policy makers on the scale of the problem in hand, and whether it’s time to intervene. It quantifies the matter of interest and gives an indication whether the intervention taken was effective or not- as a means for monitoring and evaluation [2]. Although DALY- burden of diseases metric- had succeeded to put tuberculosis (TB) back on the map as a global emergency [3], it has been constructed around the notion of cost-effectiveness [4] rather than morale of treating diseases or what is right for patients. Acknowledging this fact, I personally no longer am in favor of using this metric, along with its many critics [ibid]. In the quest of searching for a more suitable metric, it is noticeable that majority of health economic studies and reports are spinning around “economic models” stemming from the notion of “cost-effectiveness” rather than a global- equal delivery of quality healthcare.

## Main text

There is an increasing need for a “value-based” metric that assesses the ultimate outcome, rather than a “volume-based” economic metric; one that focuses on a true, measurable health outcome rather than quantifying the treatment provided. This metric must measure the effectiveness of the program rather than its efficiency.

What I am advocating for is a metric that can effectively assess the true burden of CA-MRSA, and other similar infectious diseases, away from any economic consideration. Although this might not sound “rational” to many health economics, but it rather dictates what is *right* when dealing with the problem. The proposed metric must take into consideration four variables that are directly connected to CA-MRSA:

1. Prevalence of CA-MRSA nasal colonization in a community
2. Prevalence of CA-MRSA infections
3. Incidents of CA-MRSA infections
4. Mortality linked directly to CA-MRSA infections

An effective metric that considers all those variables must inform decision makers on the following:

1. If CA-MRSA infection is under control
2. If CA-MRSA colonization is progressing rapidly into infections
3. If the population is contracting CA-MRSA infections at a rapid rate
4. If CA-MRSA infections are fatal
5. If CA-MRSA nasal colonization is declining

While this metric is not constructed yet, I believe it is a call for academics and healthcare community to consider its formatting. Further, an innovative metric might require an epiphany that might only be found at industries other than healthcare, which prompts collaboration from multiple disciplines.

Challenges to the proposed metric criteria would be convincing the policy-making mindsets that have been accustomed to concepts of economics and cost-effectiveness. Organizations are usually accustomed to their ‘business as usual’; therefore, disruptive programs might face resistance at the beginning while ignoring scientific facts. It is what I call “political momentum” by key influencers, that is required to advocate for the superiority of this strategy of concurring CA-MRSA over cost-effectiveness approaches.

## Conclusion

The use of DALY is not accurate in the context of CA-MRSA, due to the fact that majority of the cases are nor fatal neither causing major disabilities other than a nasty skin infection. On the other hand, the remaining fatal cases of CA-MRSA, anecdotally, are not expected to add on DALY. Therefore, there is a true need to identify and construct a new metric that serves the global delivery of treating and eradicating all CA-MRSA cases, as well as other similar infectious diseases, and at the same time ensures sustainability of its approach.

## Data Availability

There are no data generated from this work.

## Abbreviations

CA-MRSA: Community-acquired Methicillin-resistant Staphylococcus-aureus
DALY: Disability-adjusted Life Year.
